# Effects of Twin Inclined Plane Device on Adaptation and Ultrastructure Variations in Condyle of Growing Rats

**DOI:** 10.1155/2019/3069347

**Published:** 2019-11-14

**Authors:** Shuo Wang, Yuhong Sun, Lulu Xia, Hanyue Li, Yan Xu, Xianming Hua

**Affiliations:** ^1^The State Key Laboratory Breeding Base of Basic Science of Stomatology (Hubei-MOST), Key Laboratory of Oral Biomedicine Ministry of Education, School and Hospital of Stomatology (KLOBME), Wuhan University, Wuhan, China; ^2^Department of Orthodontics, Hospital of Stomatology, Wuhan University, Wuhan, China

## Abstract

**Objective:**

This study investigates the effects of using a twin inclined plane device (TIPD) on the remolding and ultrastructure variation of mandibular condyle in growing rats.

**Materials and Methods:**

Forty-eight male Wistar rats (six weeks old, body weight of approximately 190–210 g) were divided into experimental group (wearing appliance, *n* = 32) and control group (no appliance, *n* = 16). Samples were collected on days 3, 14, 30, and 60. The immunohistochemical analysis for vascular endothelial growth factor (VEGF) and type II collagen was carried out. Tartrate-resistant acid phosphatase (TRAP) reaction was performed to evaluate the osteoclastic activity. Three-dimensional morphometric images were reconstructed for morphometric analysis by microcomputed tomography (micro-CT). The ultrastructure of the condylar surface was observed by scanning electron microscopy (SEM).

**Results:**

The expression of VEGF significantly increased, while the expression of type II collagen decreased in the experimental group at days 30 and 60. Furthermore, the enhanced osteoclast activity was observed under the subchondral bone, which was highest at day 30, and decreased to the lowest at day 60 in the experimental group. In addition, adaptive subchondral bone remolding in the posterior part of the condyle was observed at day 60 in the experimental group, and the SEM revealed the ultrastructure variations after installation of the TIPD. However, these changes began to reverse after 30 days.

**Conclusion:**

Condylar tissue changes point to the osteoclastic activity in the posterior region of the condyle. These adaptive changes point to bone resorption in the posterior condyle. Type II collagen and VEGF contribute to the MCC remolding induced by the TIPD. The ultrastructural changes in the posterior condylar area in response to mechanical stresses are recoverable at the initial stage.

## 1. Introduction

The mandibular condylar cartilage (MCC) has a unique structure that comprises of several cartilage layers (fibrous, proliferative, mature, and hypertrophic zones) from the articular surface to the underlying bone [1]. Furthermore, the MCC is classified as an articular cartilage that has notable uniqueness, such as the capability of adaptive remolding in response to external cues during or after natural growth [2]. In terms of the remodeling of the MCC in response to mechanical loading, neovascularization plays a vital role during the adaptive changes [3–6]. Vascular endothelial growth factor (VEGF) has been shown to have overlapping functions in supporting the resorption and remodeling of subchondral bones. VEGF is produced by multiple cells, including chondrocytes, and is responsible for the migration, differentiation, and stimulation of preosteoclasts and osteoclasts into cartilaginous tissues [1]. Type II collagen forms the main collagenous framework of a cartilage, and mesenchymal cells start expressing type II collagen during the differentiation of chondroblasts [7]. Hence, the expression of type II collagen can be used as a marker for the identification and evaluation of adaptive changes in the cartilage in response to mechanical forces. In addition, mechanical overloading has profound effects on the ultrastructure changes on the condylar surface [8, 9].

Mandibular prognathism is a condition due to the excessive skeletal growth of the mandible [10]. The management of mandibular prognathism remains challenging [11, 12]. Animal models of mandibular retrusion have been far less reported in the literature, when compared to the animal models of mandibular advancement. The appliance is usually used for mandibular retraction, which applies “orthopedic forces” rather than “functional forces.” For instance, incisor guiding appliances that bonds to a rat's maxillary incisor move the mandible 3.5–4.0 mm backward, and this resulting force may harm the condyle [13]. Due to the special structure of the human temporomandibular joint (TMJ), a limited degree of retraction can be considered. Hence, incisor guiding appliances may not be useful for the treatment of mandibular prognathism. Therefore, our team designed a newly developed twin inclined plane device (TIPD), which can be placed on the upper and lower posterior tooth areas. Under rat's normal chewing behavior, TIPD can successfully induce the posterior movements of the mandible. In addition, it was found that the posterior margin of the condyle was significantly flattened after the TIPD treatment. It was speculated that TIPD can cause persistent remolding in the posterior region of the condylar cartilage [14]. However, the condylar changes in rats, which trigger the mechanism, remain unclear. Thus, exploring the effects of TIPD on the condyle would be of great interest. Therefore, the aim of the present study was to investigate the effects of using a TIPD on the remolding and ultrastructure variation of mandibular condyles in growing rats.

## 2. Materials and Methods

The present study was conducted using male Wistar rats. The study protocol and all animal experimental procedures were approved by the Ethics Committee for Animal Research, School and Hospital of Stomatology, Wuhan University, Wuhan, China.

### 2.1. Animal Experiments and Tissue Processing

**Figure 1:**
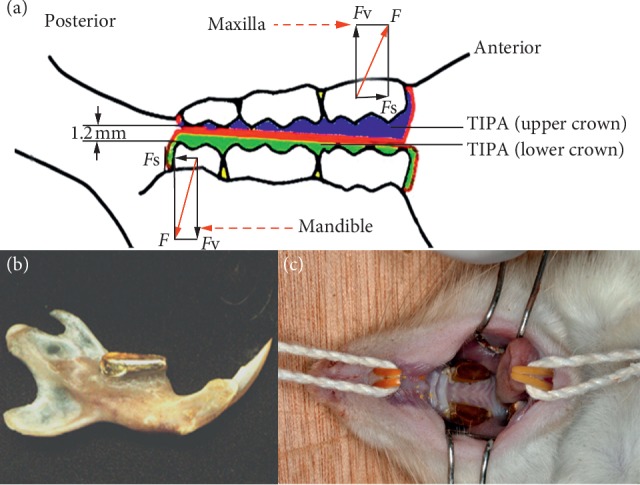
Schematic presentation of the TIPD. (a) The TIPD consists of upper and lower planes covering the posterior teeth. *F* represents the total chewing force, *F*s represents the horizontal component of *F*, and *F*v represents the vertical component of *F*. (b) The lateral view of the lower jaw installing the TIPD. (c) The TIPD was fitted to the rat posterior teeth.

Forty-eight male Wistar rats (six weeks old, body weight of approximately 190–210 g) were divided into experimental group (wearing appliance, *n* = 32) and control group (no appliance, *n* = 16). All animals were regularly provided with food and water. All TIPD appliances used in the present study were fabricated using alloy (Degussa Dental, Hanau, Germany) and a casting machine (Degussa Dental, Hanau, Germany). In order to fabricate the TIPD, a rat skull we first prepared, and the impression trays were tailored to obtain the model of the upper and lower dentition of rats. Then, rats in the experimental group were anaesthetized, and the impressions were obtained using a silicone rubber poured with super-hard gypsum. Afterwards, the TIPD was fabricated using the lost wax and casting technique. In the experimental group, the upper and lower teeth of rats were acid-etched, and the TIPD was bonded to the rat's posterior teeth using GC (Fuji Ortho LC, Tokyo, Japan; Figure 1). Any high bite points detected on the TIPD were adjusted using a portable turbine, until the upper and lower planes are in close contact. In the present study, the TIPD was bonded with natural teeth and examined on regular a basis. It was found that the appliance of one rat fell off on the 2^nd^ day and the appliance of two rats fell off on the 5^th^ day of the experiment. All detached appliances were rebounded using the acid-etching technique, as earlier described. Eight rats in the experimental group and four rats in the control group were euthanized by anesthesia using pentobarbital (200 mg/ml; Alfasan, Netherlands) after 3, 14, 30, and 60 days, respectively. The soft tissues from the head were removed and fixed using 4% paraformaldehyde for 48 hours, and the heads were dissected along the sagittal plane. The left halves were harvested for immunohistochemical staining, while the right halves were used for microcomputed tomography (micro-CT) and scanning electron microscopy (SEM) analysis. In the experimental group, the eight right condyles were divided into two groups (four in each group) and were examined by SEM and micro-CT, respectively.

### 2.2. Histological and Immunohistochemical Staining

Immunohistochemical staining was performed to detect the VEGF and type II collagen levels. After deparaffinization in xylene, dehydration in ethanol and washing with phosphate-buffered saline (PBS, pH 7.4) were performed. Then, the sections were antigen-retrieved by pepsin and incubated with 0.3% hydrogen peroxide (20 minutes) to block the endogenous peroxidase activity, followed by processing with serum (30 minutes) to block the unspecific ligations. Immunohistochemical staining was performed for sections incubated with the following antibodies:

1. Rabbit anti-VEGF (1 : 100; sc-30044, Santa Cruz Laboratories, Santa Cruz, CA, USA)
2. Rabbit anti-COL II (1 : 100; sc-7763, Santa Cruz Laboratories, Santa Cruz, CA, USA) for 24 hours at 4°C

Next, the sections were washed and incubated using an immunohistochemical kit (Zhongshan Biotechnology Co., Ltd., China) and visualized by 3,3-diaminobenzidine tetrahydrochloride (DAB). Finally, the sections were counterstained with hematoxylin. The tartrate-resistant acid phosphatase (TRAP) reaction was also objected to assess the osteoclast number.

### 2.3. Microcomputed Tomography (Micro-CT) Scanning

In order to evaluate the bony changes in the condyle, the samples were scanned by micro-CT (*μ*CT50, Scanco Medical, Bassersdorf, Switzerland) at a slice thickness of 15 *μ*m, and the morphometric analysis was performed by three-dimensional reconstruction.

### 2.4. Quantitative Analysis

**Figure 2:**
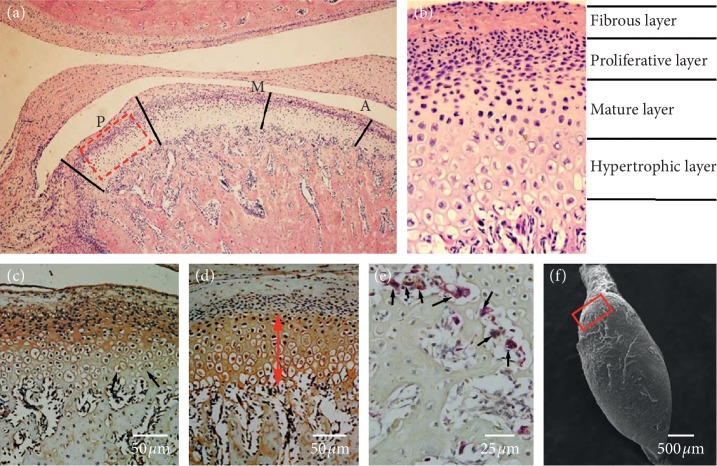
The MCC structure and related factors detected in the present study. (a) The mandibular condyle cartilage (H&E, ×50) was divided into three regions: anterior (A), middle (M), and posterior (P). The red dotted box indicates the MCC district of 1044 × 766. (b) The basis of the present regions of MCC. (c) The black arrow reveals the VEGF positive cells. (d) The type II collagen layers (red arrows). (e) Osteoclast (black arrow). (f) The SEM image of the mandibular condyle. The red box indicates the posterior surface area of interest.

The present study divided the surface of the condylar cartilage into three equal parts: anterior, middle, and posterior (Figures 2(a) and 2(b)). TIPD can gradually induce the mandibular backward movements and change the centric occlusal relationship of the rat's condyle to the retreated position. The horizontal component of the chewing force, *F*s, is transmitted to the posterior region of the condyle (Figure 1(a)). Therefore, the posterior region was determined as the focus of the present study [14]. The expression of VEGF (Figure 2(c)), type II collagen (Figure 2(d)), and osteoclasts (Figure 2(e)) was semiquantified using an Olympus DP72 microscope (Olympus DP72 microscope, Olympus Corporation, Japan) and the Image-pro plus 6.0 software (Media Cybernetics, USA).

### 2.5. Scanning Electron Microscopy

The samples were fixed using glutaraldehyde (25 g/L) at 4° for 48 hours. Then, the samples were rinsed using phosphate buffer (0.1 mol/L) and refixed in osmic acid (10 mol/L). Upon drying at the critical point and vacuum spraying, the samples were observed by scanning electron microscopy (VEGA3, TESCAN, CZ; Figure 2(f)).

### 2.6. Statistical Analysis

The posterior condylar area was selected to cover three consecutive regions of the fibrous layer, proliferative layer, mature layer, and hypertrophic layer (1044 × 766 pixels). The relative area of positive staining of VEGF, the thickness of the type II collagen layer, and the number of osteoclasts were calculated. Repeated experiments in the same manner confirmed the reproducibility of these results. All data were expressed as mean ± standard deviation. GraphPad Prism 6.0 (GraphPad Software, USA) was used to perform the statistical analysis with one-way analysis of variance (ANOVA) and the Tukey–Kramer multiple comparisons test. The resulting *P* value of <0.05 was considered statistically significant, while a *P* value of <0.01 was considered as highly statistically significant.

## 3. Results

### 3.1. Effects of the TIPD on VEGF and Type II Collagen in the Posterior Region of the Condyle

**Figure 3:**
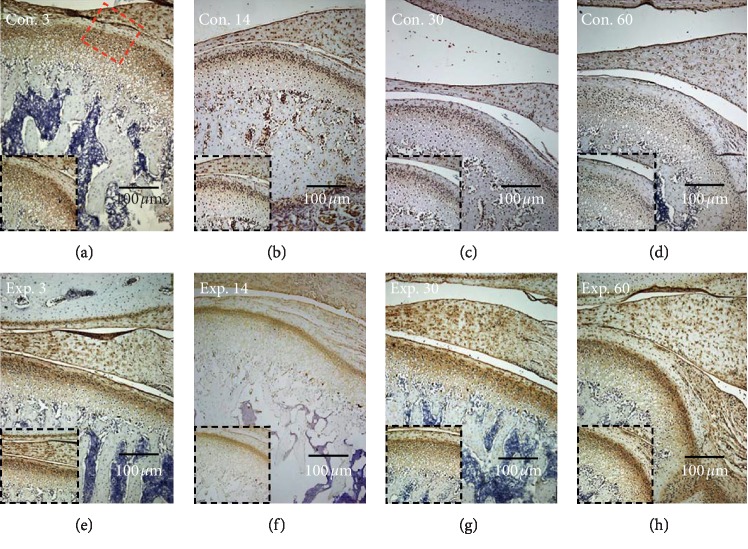
The TIPD induced the expression of VEGF in the posterior region of the condyle. (c, d) The expression of VEGF was weak in the control day 30 and control day 60 groups, (g, h) but this increased from 30 to 60 days in the experimental group. The red box indicates the area of interest, and this is shown in the black box area.

**Figure 4:**
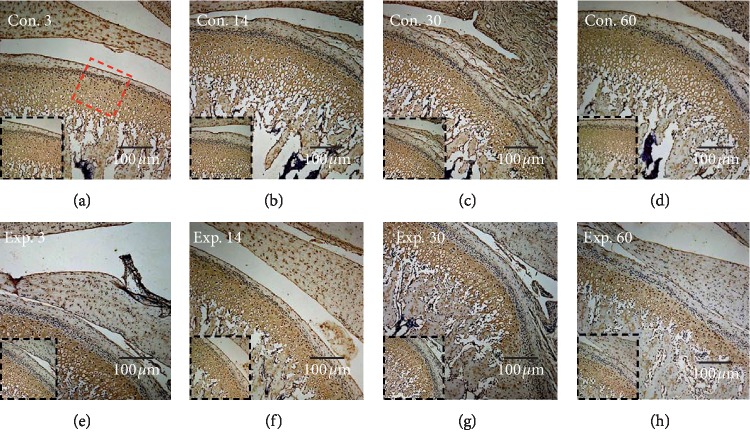
The TIPD inhibited the expression of type II collagen in the posterior region of the condyle. (a–d) The expression of type II collagen was stronger in the control group, (g, h) but this decreased from 30 to 60 days in the experimental group. The red box indicates the area of interest, and this is shown in the black box area.

In growing rats, the mechanical forces produced by mandibular backward movement led to an increased expression of VEGF (Figure 3) and a decreased expression of type II collagen (Figure 4), when compared to the control group.

**Figure 5:**
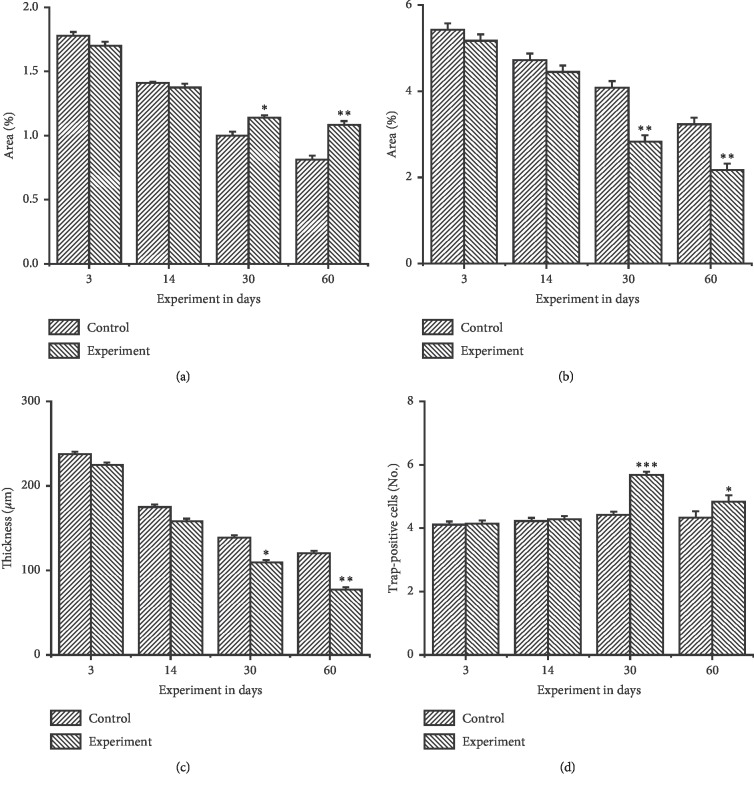
Analysis of the expression of VEGF, Type II collagen, and osteoclasts in the posterior condylar area. (a) The VEGF expression in the posterior part of the condyle in the control group and experimental group. The significant difference was annotated on days 30 and 60 between the experimental and control groups (^*∗*^*P* < 0.05, ^*∗∗*^*P* < 0.01). (b) The expression of type II collagen decreased in the posterior part of the condyle within 30–60 days in the control and experimental groups. A significant difference was annotated on days 30 and 60 (^*∗*^*P* < 0.05, ^*∗∗*^*P* < 0.01). (c) The thickness of the mature and hypertrophic layer presented by type II collagen in the posterior part of the condyle in the control and experimental groups. A significant difference in thickness was observed in the control and experimental groups at day 30 and 60 (^*∗*^*P* < 0.05, ^*∗∗*^*P* < 0.01). (d) TRAP-positive cells were observed under the subchondral bone in the control and experimental groups. Significant differences in the number of osteoclasts in animals in the experimental and control groups were observed at day 30 and day 60 (^*∗*^*P* < 0.05, ^*∗∗*^*P* < 0.01, ^*∗∗∗*^*P* < 0.001).

Compared with the control group, the expression of VEGF was increased significantly from day 30 to day 60 and the highest level was achieved on the day 60 (Figure 5(a)), while the expression of type II collagen in the experimental group decreased from day 30 and the lowest level was achieved on day 60 (Figure 5(b)). The thickness of the type II collagen positive layer (mature and hypertrophic layer) exhibited a significant decrease in the experimental group at days 30 and 60 (Figure 5(c)).

### 3.2. Effects of the TIPD on TRAP-Positive Cells in the Posterior Region of the Condyle

**Figure 6:**
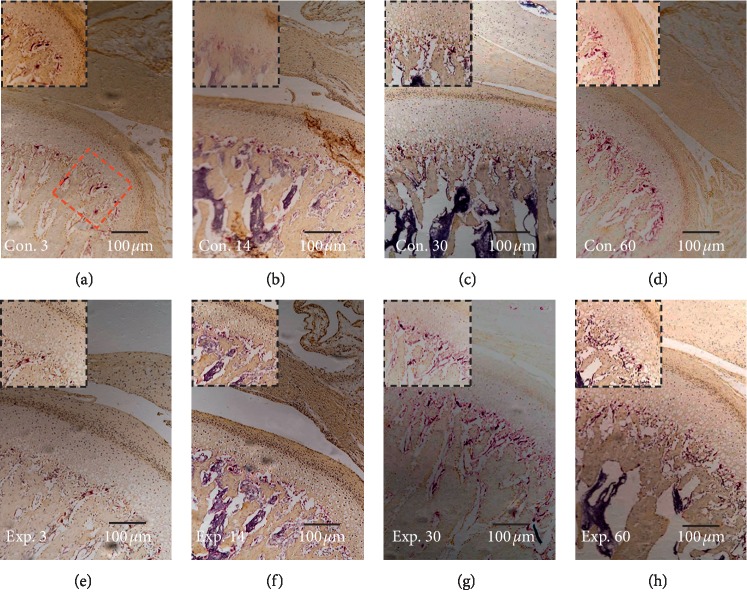
The TIPD increased the expression of osteoclasts in the posterior region of the condyle. The presence of osteoclasts and osteoclastic activity in the experimental group are shown. (g) At day 30, there was a significant increase in osteoclasts in the region between the MCC and endochondral bone and this decreased to the lowest on day 60 (h). The red box indicates the area of interest, and this is shown in the black box area.

TRAP-positive cells were found between the MCC and subchondral bone (Figure 6). The number of osteoclasts increased on day 30 and decreased to the lowest level on day 60 in the experimental group (Figure 5(d)). However, this remained significantly (*P* < 0.05) higher, when compared to the control group.

### 3.3. Adaptive Subchondral Bone Remolding in the Posterior Part of the Condyle

**Figure 7:**
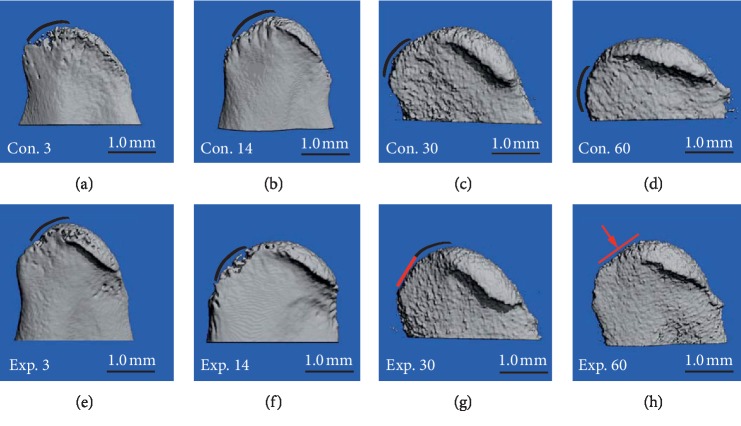
The 3D images of the posterior margin of the condyle's lateral view in growing rats in control and experimental groups. (a–f) The condyle in the control group and experimental group at days 3 and 14 all had a smooth and circular arc. (g) For condyles in the experimental group at day 30, the lower part of the posterior margin of the condyle began to flatten. (h) At day 60 in the experimental group, the posterior margin of the condyle was completely flattened.

In the control group, the posterior margins of the condylar subchondral bone were round (Figures 7(a)–7(d), black arc line). In the experimental group at day 30, the lower part of the posterior margin of the condyle (Figure 7(g), red straight line) began to flatten, but the superior part remained arc shaped (Figure 7(g), black arc line). In the experimental group at day 60, the entire posterior margin of the condyle appeared to be significantly flattened (Figure 7(h), red straight line).

### 3.4. Ultrastructure Variations after Installation of the TIPD

**Figure 8:**
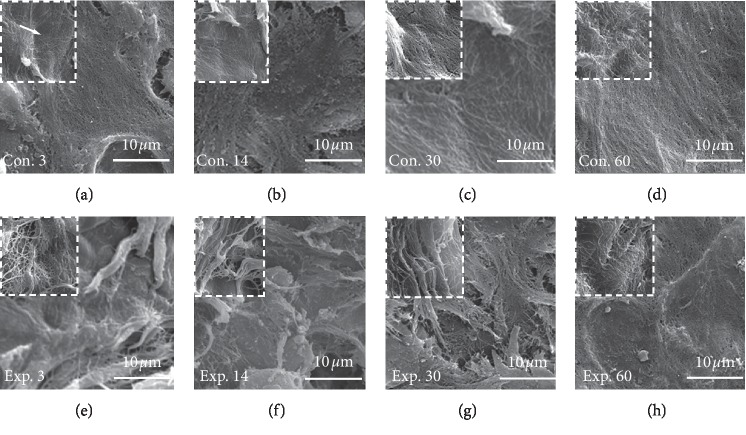
The SEM images (magnification ×4,000; white dotted box ×10,000) of the posterior area of the condylar surface. The condylar surface of the control group shows a shallow wavy appearance and uniformly distributed collagen fibers. (e, f) The condylar surface in the experimental group at days 3 and 14 shows that the gel layer flaked off, and the internal collagen net was exposed with evulsion of the collagen fibers. (g) The condylar surface in the experimental group at day 30 shows that the changes in the condylar surface began to reverse, and tissue repair was observable around the altered areas. (h) The condylar surface in the experimental group at day 60 shows the repair processes, in which the fibers were slowly surrounded by the matrix, and the disorganized collagen fibers on the surface were less than those in the experimental group at day 30.

In addition, the ultrastructure of the condylar surface was compared between the control and experimental groups (Figure 8). The condylar surface in the control group had a shallow wavy appearance, along with uniformly distributed collagen fibers (Figure 8(a), white arrow). In the experimental group at day 14 (Figure 8(f)), the deep condylar collagen was exposed, and cracks were observed on the surface of the condyle. However, these changes began to reverse in the experimental groups from days 30 to day 60 (Figures 8(g) and 8(h)).

## 4. Discussion

In the present study, an animal model was used, and the remolding and ultrastructure variations in the mandibular condylar tissues of growing rats were investigated while using TIPD. The changes in condylar tissues were evaluated in various aspects using techniques, such as immunohistochemical analysis, TRAP, micro-CT, and SEM. The present results revealed that biomechanical stresses exerted by TIPD enhanced the osteoclast activity under the subchondral bone and effected the growth of the posterior condylar region. Decrease of type II collagen and the increase of VEGF were observed during the adaption of the condylar and remodeling.

VEGF is an important mediator of neovascularization in growing rats, and its expression is upregulated with the period of mechanical stresses [15, 16]. In a previous study, Wu et al. reported that the compressive force applied to the TMJ enhanced the expression of VEGF, activating the angiogenesis [4]. Similarly, a chondrocyte culture model revealed that VEGF is significantly upregulated by hydrostatic pressure [17]. In the present study, the expression of VEGF in the posterior region of the condylar cartilage significantly increased among the experimental animals at day 30, suggesting that VEGF contributes to the adaption induced by TIPD.

Meanwhile, type II collagen, which is the main component of the organic matrix of MCC, was quantitatively assessed during the experimental period. It was observed that type II collagen continuously deceased from experimental day 30 to day 60 under the gradual backward movement of the mandible, thereby indicating that the TIPD can inhibit the proliferation of chondroblast and induce the remolding of the posterior condylar cartilage. These findings are in agreement with those of the previous studies [18, 19]. In the model of applying high-intensity stresses on a rat's intervertebral disk, the amount of type II collagen significantly decreased [18]. Similarly, the articular cartilage used in explant studies revealed that the static compressive overloading inhibited the expression of aggrecan and thereby downregulated the type II collagen mRNA [19].

During the growth and development of rats, VEGF is enriched in the MCC and subchondral bone, which can induce the secretion of type II collagen. Osteoclasts can absorb the calcified matrix of the cartilage to promote osteogenesis. In the control group, VEGF is mainly present in and around hypertrophic chondrocytes. Aoyama et al. [20] reported that VEGF gradually increased with age, peaked at 10–20 days, and subsequently decreased. In the present experiment, the rats were six weeks old, and the expression of VEGF gradually decreased, which was consistent with the above results. Hypoxia inducible factor-1 (HIF-1) is regulated by changes in local oxygen tension and plays an important role in the differentiation of chondrocytes and production of the extracellular matrix [21]. Applying compressive stresses to chondrocytes activates HIF-1, which in turn induces the synthesis of VEGF, thereby stimulating the production of collagen-degrading enzymes, such as metalloproteinases (MMPs) [22, 23]. In the present study, type II collagen decreased, while VEGF expression significantly increased on day 30 in the experimental group. Therefore, it can be postulated that the gradually induced backward movements of the mandible reduce the joint space between the posterior condyle and TMJ capsule. In addition, the decreased local oxygen pressure may activate the expression of HIF-1. Subsequently, HIF-1 stimulates the production of VEGF, thereby promoting MMPs to perform degradation effects on the type II collagen in the posterior condylar area.

The 3D reconstruction of the condylar head revealed the normal contour of the posterior surface in the control group, while the contour was entirely flattened in the experimental group at day 60. These findings suggest that the present TIPD animal model is reliable, and successfully induced the backward movements of the mandible. It has been demonstrated that the barrier between the cartilage and underlying bone brakes under high-intensity stresses, thereby promoting VEGF to disperse to the underlying bone region and inducing the aggregation of osteoclasts [24]. In the present study, a significant increase in osteoclastic cells was observed in the underlying bone in the experimental group at day 30 when a higher expression of VEGF was identified. The micro-CT result revealed that the experimental group began to flatten on day 30 in the experimental group, and the expression of type II collagen decreased, which may be correlated to the increase of osteoclasts. It is noteworthy that the adaption, bone remodeling, and level of bone mass reflect the balance of bone formation and resorption [25]. Unfortunately, focus was merely given on bone resorption to explain the subchondral resorption. As shown in recent researches [26, 27], osteoclast-mediated EphrinB2 can cause osteoblasts to bind to EphB4, which in turn promotes osteogenesis. TGF, OPG, and other factors that promote osteogenesis can also be found in the attachment of the osteoclast absorptive pit. Therefore, not only the osteoblast but also the bone forming mechanism of the osteoclast in the context should be carried out in further studies.

The thickness of maturative and hypertrophic layers was combined as the “type II collagen positive layer.” In an animal model of bite-jumping retrusion of the mandible, the thickness of the cartilaginous layer area in the posterior region significantly decreased in as early as seven days [28]. In addition, Kuroda et al. [29] investigated the posterior displacement of the condyle using an animal model and reported the enhanced expression of osteoclasts in as early as 14 days. However, in the present study, the significant decrease in type II collagen and increased expression of osteoclasts were observed comparatively late (at 30 days). The reason for the “delayed expression of type II collagen and osteoclasts” might be due to the mild forces induced by the countless stepwise backward movements under normal cyclic masticatory stresses, rather than the forced mandibular retrusion induced by the single-jump step, as reported by Teromoto et al. [28] Furthermore, osteoclasts in the posterior condylar region reached its peak levels on day 30 and decreased to the lowest on day 60. These changes are likely due to the adaption. That is, the condylar tissues gradually adapted to the mild and continuous force produced by the TIPD from day 30 to day 60.

The ultrastructure of the condylar surface determined by SEM revealed that the normal condyle consisted of corrugated structures and was covered by a thin layer of granular gel. In contrast, the experimental group at day 14 revealed uneven and irregular fissures on parts of the surface, and the associated collagen fibers were exposed in a disordered and loose arrangement. Under psychological stresses, the ultrastructure of the rat TMJ may be affected by the alteration of masticatory muscle functions [8, 30]. Thus, it was hypothesized that installing a TIPD on the teeth may cause discomfort and psychological stress to young rats. The ultrastructure of the condylar surface also exhibited the reversal of TMJ changes from day 30 and onwards. For instance, in the experimental group at day 60, the SEM image revealed the better organization of collagen fiber bundles, which is similar to that of the control group at day 60. These findings suggest that the adaption induced by the TIPD was physiological and that the initial damage to the condylar surface was recoverable. However, there were still differences, when compared to the control, since it is not shaped into a complete continuous gel-like membrane-like structure. Hence, it is necessary to prolong the experimental period for such observations. The strength of the mechanical stress is a crucial factor that affects the deformation of the collagen-proteoglycan network [9]. Furthermore, the strength of horizontal component forces may remarkably vary with the angle between the upper and lower posterior long inclined planes. Thus, further studies are needed to identify the specific angle that might reduce the incidence of fibrils and collagen deformation of the condylar surface.

## 5. Conclusions

Condylar tissue changes point to the osteoclastic activity in the posterior region of the condyle. These adaptive changes point to bone resorption in the posterior condyle. The type II collagen and VEGF contribute in MCC remolding during the backward movement of the mandible induced by the TIPD. The ultrastructural changes in the posterior condylar area in response to mechanical stresses are recoverable at the initial stage.

## Data Availability

The data used to support the findings of this study are available from the corresponding author upon request.
